# Revealing the Usefulness of Aroma Networks to Explain Wine Aroma Properties: A Case Study of Portuguese Wines

**DOI:** 10.3390/molecules25020272

**Published:** 2020-01-09

**Authors:** Sílvia Petronilho, Ricardo Lopez, Vicente Ferreira, Manuel A. Coimbra, Sílvia M. Rocha

**Affiliations:** 1 QOPNA & LAQV-REQUIMTE, Chemistry Department, University of Aveiro, 3810-193 Aveiro, Portugal; silviapetronilho@ua.pt (S.P.); mac@ua.pt (M.A.C.); 2 Laboratory for Flavor Analysis and Enology, Instituto Agroalimentario de Aragón (IA2), Analytical Chemistry Department, Faculty of Sciences, University of Zaragoza, E-50009 Zaragoza, Spain; riclopez@unizar.es (R.L.); vferre@unizar.es (V.F.)

**Keywords:** monovarietal wines, gas chromatography, volatile compounds, aroma network, aroma sensory analysis

## Abstract

Wine aroma is the result of complex interactions between volatile compounds and non-volatile ones and individual perception phenomenon. In this work, an aroma network approach, that links volatile composition (chromatographic data) with its corresponding aroma descriptors was used to explain the wine aroma properties. This concept was applied to six monovarietal wines from Bairrada Appellation (Portugal) and used as a case study. A comprehensive determination of the wines’ volatile composition was done (71 variables, i.e., volatile components), establishing a workflow that combines extraction techniques and gas chromatographic analysis. Then, a bipartite network-based approach consisting of two different nodes was built, one with 19 aroma descriptors, and the other with the corresponding volatile compound(s). To construct the aroma networks, the odor active values were calculated for each determined compound and combined with the bipartite network. Finally, the aroma network of each wine was compared with sensory descriptive analysis. The analysis of the specific aroma network of each wine revealed that Sauvignon Blanc and Arinto white wines present higher fruity (esters) and sweet notes (esters and C_13_ norisoprenoids) than Bical wine. Sauvignon Blanc also exhibits higher toasted aromas (thiols) while Arinto and Bical wines exhibit higher flowery (C_13_ norisoprenoids) and herbaceous notes (thiols), respectively. For red wines, sweet fruit aromas are the most abundant, especially for Touriga Nacional. Castelão and Touriga Nacional wines also present toasted aromas (thiols). Baga and Castelão wines also exhibit fusel/alcohol notes (alcohols). The proposed approach establishes a chemical aroma fingerprint (aroma ID) for each type of wine, which may be further used to estimate wine aroma characteristics by projection of the volatile composition on the aroma network.

## 1. Introduction

The concept of wine quality is very complex, being related with several intrinsic factors (defined by the drinking experience, namely pleasure, aroma, flavor, color, and mouthfeel) as well as extrinsic ones (winemaking, price, origin, bottle form, and color) that influence consumers’ choices [1,2]. From these, the aroma is considered one of the most significant factors to establish wine quality and character and, therefore, for determining consumers’ acceptance. A relationship between wine character and its volatile composition is recognized by several researchers worldwide, and hundreds of volatile compounds belonging to different chemical classes, namely alcohols, esters, acids, aldehydes, ketones, lactones, terpenoids, and volatile phenols, have already been identified in different wine varieties [3,4,5,6,7,8,9]. As these compounds produce an effect on consumers’ sensory perceptions, both volatile composition and sensory properties are essential to determine wine aroma characteristics.

Gas chromatography (GC)-based techniques provide an effective tool for the determination of wine volatile compounds. However, knowledge about the wine volatile composition alone is insufficient to explain or predict the whole wine aroma properties [10,11]. Several reports have attempted to model sensory properties from wine chemical data by using multivariate statistical techniques. For instance, to predict the sensory attributes of Cabernet Sauvignon [12] and Albariño wines [13], partial least squares (PLS) regression was used. In addition to the fact that several volatile components revealed to be important to the sensory attributes modeling, these observations are limited to the number of samples used from a single variety, being necessary for the analysis of additional wines that share the reported sensory characteristics. Moreover, analysis of variance (ANOVA) [14] was also applied to assess the aroma vectors that influenced the perception of descriptors in model red wines. However, from the six vectors studied only four major red wine sensory notes were satisfactorily reproduced and this was not performed in real wine samples, thus it did not consider all matrix interferents. Thus, due to the high economic value of the wine-product worldwide, the exploitation of innovative approaches, which combine wine volatile composition (instrumental data) with their corresponding aroma descriptors, to explain wine aroma properties, is extremely important. The network-based approach seems to be a potential tool. This was introduced as a network that captures the flavor compounds shared by culinary ingredients, exploring the impact of these compounds on ingredient combinations [15]. Firstly, a bipartite network with two different nodes is built, with one node as the aroma descriptor, and the other as related volatile compounds. The result of the combination of this bipartite network with the odor active values (OAVs, described as the ratio between the concentration of each volatile component in a wine sample [16]) of each volatile is an aroma network that allows to understand and/or estimate the potential contribution of each compound for aroma properties. In this aroma network, two nodes are linked if they share at least one aroma descriptor, and the thickness of the line used between aroma descriptors is proportional to the number of shared compounds [15]. Recently, the bipartite network approach was successfully applied to explain two aroma classes of lager beers [17].

The present research study aims to apply the aroma network concept to explain the aroma properties of white and red wines. For this, several chemical families, known to contribute to wine aroma perceptions, namely esters, carbonyl compounds, alcohols, acids, terpenic compounds, lactones, C_13_ norisoprenoids, volatile phenols, and thiols, were considered. Monovarietal commercial wines from Bairrada Appellation were selected as a case study, produced from five autochthonous varieties (Arinto, Bical, Baga, Castelão, and Touriga Nacional) and a worldwide cultivated one (Sauvignon Blanc). For the construction of the aroma network, in the first step, a detailed determination of the wines’ volatile compounds was done to establish a workflow that combines extraction techniques and GC analysis. Then, a bipartite network-based approach was built consisting of two nodes: the 19 aroma descriptors and the corresponding volatile compound(s). To construct the aroma network for each wine variety, the OAVs were calculated for each determined compound, summed together based on their aroma categories, and combined with the bipartite network. The differences in the OAV proportions among the aroma descriptor categories were then compared to descriptions from the sensory analysis. The proposed approach disclosed that the construction of the aroma networks is useful to explain wine aroma peculiarities, based on the combination of chemical and sensorial data. The six monovarietal wines, including three white and three red wines, with distinct chemical and sensorial characteristics, allowed to obtain results that are appropriate for the proof-of-concept of the aroma network strategy under study.

## 2. Results and Discussion

### 2.1. Volatile Components Determination by GC-Based Techniques

A total of 71 volatile compounds, distributed over nine chemical families, including esters, alcohols, acids, carbonyl compounds, terpenic compounds, C_13_ norisoprenoids, lactones, phenols, and thiols, were determined in the wines under study (Tables S1 and S2 for white and red wines, respectively). In addition to their concentration, the already known odor threshold values (the lowest concentration of a compound in vapory phase which can be detected by smell) [18,19] and the OAVs for each determined compound were also considered (Tables S1 and S2).

**Figure 1:**
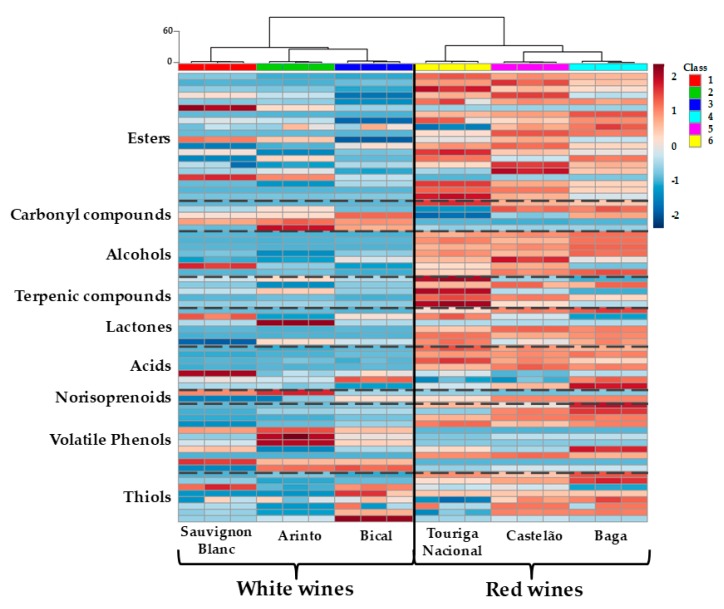
Heatmap and dendrogram representation corresponding to the 71 volatile compounds of the six *Vitis vinifera* L. monovarietal wines under study: Sauvignon Blanc (class 1, red), Arinto (class 2, green), Bical (class 3, dark blue), Baga (class 4, blue), Castelão (class 5, purple), and Touriga Nacional (class 6, yellow). The relative content of each compound is illustrated through a chromatic scale (from dark blue, minimum, to dark red, maximum). Dendrogram for the HCA (hierarchical cluster analysis) results, using Ward’s cluster algorithm for the data set, was also included. Detail data are reported in Tables S1 and S2 for white and red wine varieties, respectively.

To make an easier analysis of the data set concerning the volatile pattern of the six monovarietal wines, a heatmap representation was performed (Figure 1), corresponding to the graphical representation of the data from Tables S1 and S2. The heatmap allows rapid visual access of each wine volatile profile and the relative comparison of the six monovarietal wines. The chromatic scale of the heatmap accesses the relative amount of each volatile compound (from dark blue, minimum, to dark red, maximum) and consequently, to observe the chemical family pattern for the wines under study. Whereas the dendrogram (Figure 1) built from the HCA (Hierarchical Clustering Analysis) is an exploratory tool that reveals clustering between the wines under study, gathering them according to their volatile profile’s similarities. According to this, for each wine studied, it was possible to observe the formation of six clusters: Sauvignon Blanc (class 1, red), Arinto (class 2, green), Bical (class 3, dark blue), Baga (class 4, blue), Castelão (class 5, purple), and Touriga Nacional (class 6, yellow). Furthermore, despite all the variables among these wines, it was also possible to observe the formation of two main clusters (Figure 1), corresponding to the white (classes 1, 2, and 3) and the red wines (classes 4, 5, and 6) under study.

The heatmap shows that although the identified chemical families are the same for all wines under study, the volatile composition determined for white and red wines is different, with the red wines having greater amounts of almost all the determined volatile compounds (Figure 1), which was in line with previously reported works [20,21]. The most abundant red wine components are essentially esters, alcohols, and acids (mainly C_4_–C_10_ fatty acids). These compounds, produced during alcoholic fermentation, play an important role in wines’ aroma [22,23]. Covering all the identified chemical families, esters represent the largest one (a total of 21 compounds were determined). These compounds were already described as important compounds in young wine aroma, associated with their fruity and sweet notes [19,24,25].

Considering all wines studied, Bical presents the lowest content of esters, while Touriga Nacional presents the highest content (Figure 1). On the other hand, alcohols and acids are quantitatively the largest groups of volatile compounds determined, although their composition is different between the wine varieties: a higher amount was determined for red varieties, principally Baga red wine, while a lower amount was found for Arinto white wine. Among these, higher amounts of isoamyl alcohol, phenylethanol, isobutanol, and acetic acid were determined. Quantitatively, isoamyl alcohol accounts for more than 50% of all alcohols determined in all the red wines studied, being higher in Baga and lower in Touriga Nacional wines. Isoamyl alcohol is considered the main aliphatic alcohol synthesized by yeast during fermentation, comprising 40–70% of the total alcohol fraction [22]. Furthermore, the C_6_ alcohols, such as 1-hexanol and (*z*)-3-hexen-1-ol, recognized as having a negative effect on wine aroma when their concentrations are above their odor threshold values [26], were determined at concentrations below their odor threshold values in all the analyzed wines (Tables S1 and S2). Moreover, the presence of benzyl alcohol and phenylethanol aromatic alcohols is associated with positive descriptors, and these were determined in all varieties studied, suggesting their contribution to the aroma characteristics of these wines. In all varieties, phenylethanol was above its odor threshold level (14 mg/L). This compound was already quantified in Bical [27] and Sauvignon Blanc wines [28], and similar amounts were determined (ca. 16 and ca. 18 mg/L for Bical and Sauvignon Blanc, respectively) when compared with those previously reported (ca. 20 mg/L and 17–43 mg/L, respectively). A total of six lactones were also identified and quantified in the studied monovarietal wines. The most abundant lactone is γ-butyrolactone, principally in the red wines, ranging from ca. 8.2 mg/L in Touriga Nacional wine to ca. 16.6 mg/L in Baga wine (Table S2). Furthermore, carbonyl compounds, which are related to young white wine oxidation [29], prevail in white wines, principally in Arinto and Bical.

Other important compounds, such as volatile phenols, have been determined. 4-Ethylphenol, 4-ethylguaiacol, and 4-vinylphenol were the most abundant volatile phenols determined in the white wines, principally in Arinto wine, while acetovanillone was the most abundant for the red wines. Although volatile phenols can contribute positively to the aroma of wines, they are better known for their contribution to off-flavors such as animal or leather notes, resulting essentially from high concentrations of ethylphenols. For instance, at concentrations above 1.74 mg/L, 4-ethylphenol is reported as presenting a negative contribution to the aroma [30]. However, as the maximum concentration of this compound in these set of wines was ca. 0.6 mg/L in Arinto wine, this off-flavor was not expected.

Varietal compounds, including C_13_ norisoprenoids and terpenic compounds, were also determined. Two C_13_ norisoprenoids were quantified: β-damascenone and β-ionone. β-Damascenone was found at a higher amount, mainly in Arinto and Sauvignon Blanc white wines, and Touriga Nacional red wine. This compound is reported as having a determinant role in the Arinto wine aroma profile [31]. Among the quantified monoterpenic compounds, linalool, α-terpineol, and geraniol were the most abundant. The level of monoterpenols was higher in Touriga Nacional wine than in the other red wines studied (Figure 1). In fact, wines from Touriga Nacional are considered richer in terpenol compounds [32]. From the white wines studied, the lowest amount of terpenic compounds was found in Bical wine (Figure 1, Table S1). The terpenic profile of Bical wine was already quantitatively determined and this wine was found to be teeming with terpenic compounds [27]. Furthermore, the major monoterpenols determined in Arinto wine were linalool and α-terpineol, which follows the results of Rocha et al. [31].

Regarding the volatile thiols, methionol, 2-methyl-3-furanthiol, and 3-mercapto-1-hexanol were the most abundant thiols found in the studied Bairrada wines, in agreement with the data reported for Zalema wines from Spain [19]. For white wines, Bical and Sauvignon Blanc exhibited higher amounts of these compounds and for the red wines, a lower amount was determined in Touriga Nacional. These compounds are already considered important aroma compounds in Sauvignon Blanc wines [28].

According to these results, the volatile profiles determined for the wines under study (Figure 1) can contribute to the distinction of these varieties based on their different chemical compounds, amount, and composition. The detailed knowledge of each wine volatile composition is essential to explain the differences between their aroma properties.

### 2.2. Aroma Network Approach for Wine Aroma Fingerprint

#### 2.2.1. Wine Aroma Network Construction

**Figure 2:**
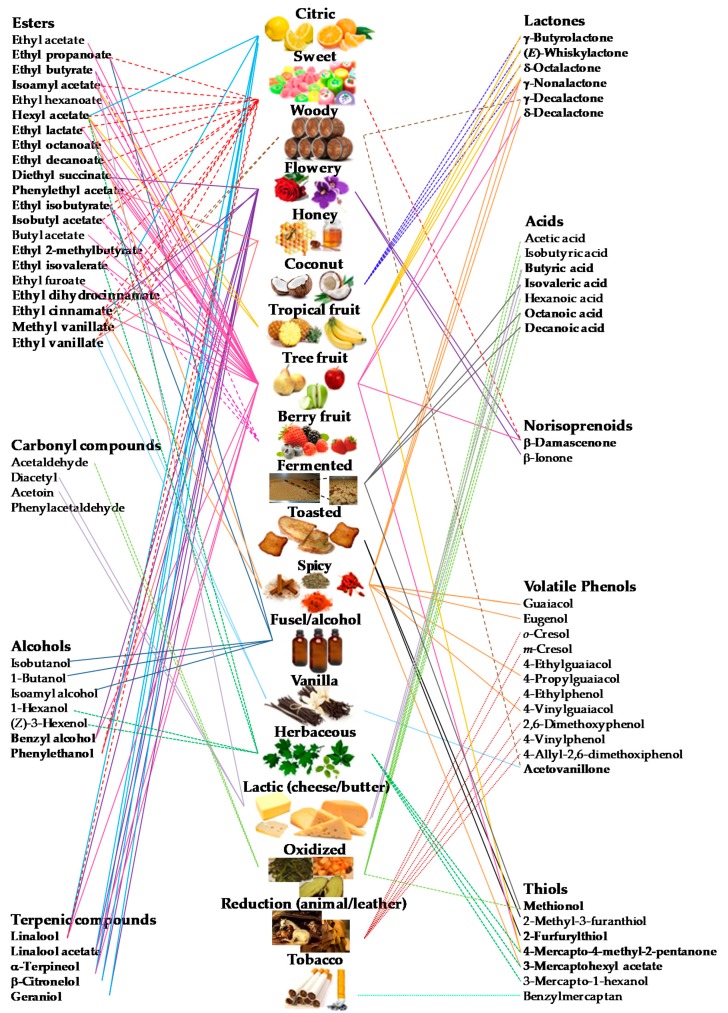
Proposed bipartite network-based approach to explain wine aromas, constructed using the volatile composition of six white and red wines. The central column represents the 19 aroma descriptors under study and the lateral ones correspond to the 71 volatile compounds that may contribute to those notes. Bold names indicate the volatile compounds that shared at least two or more aroma descriptors.

For the construction of the aroma network for the six monovarietal wines under study, a comprehensive determination of each wine volatile composition was done establishing a workflow that combines extraction techniques and GC analysis. Then, to explain the wine aroma properties, a bipartite network-based approach consisting of two different nodes was built (Figure 2): the central column represents the 19 aroma descriptors under study, namely citric, sweet, woody, flowery, honey, coconut, tropical, tree and berry fruits, fermented, toasted, spicy, fusel/alcohol, vanilla, herbaceous, lactic (cheese, butter), oxidized, reduction (animal, leather), and tobacco; and the lateral ones correspond to the 71 volatile compounds that may contribute to those notes. Figure 2 reveals that the aroma of the studied wines is very complex, with several volatile compounds sharing at least two or more aroma descriptors (represented in bold), which explains the different wines’ aroma properties.

**Figure 3:**
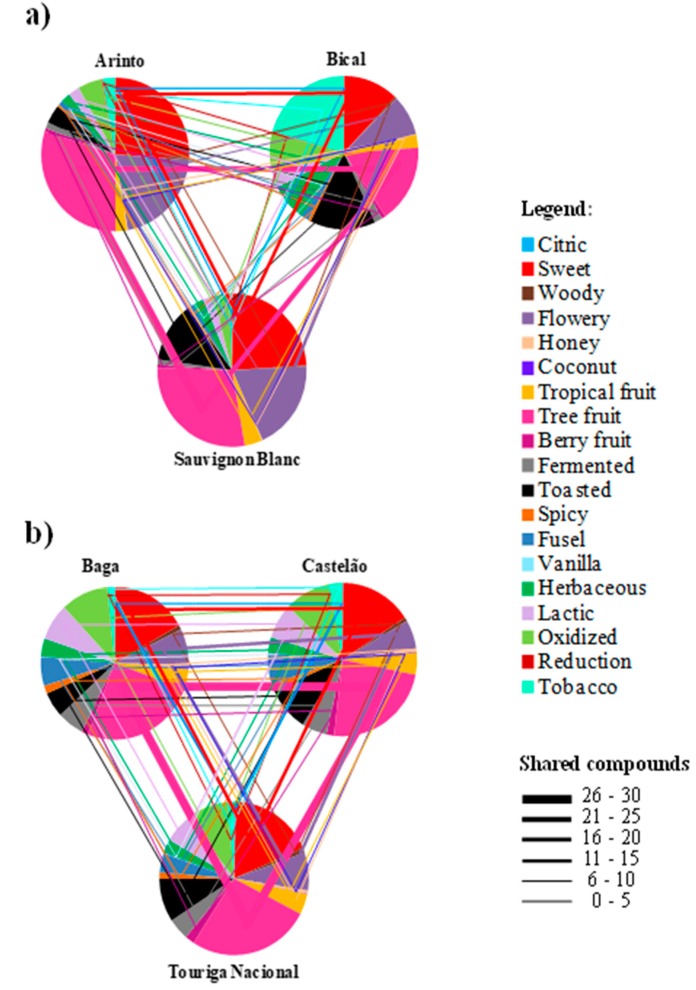
Aroma networks constructed for the six wines under study, based on the bipartite network: (**a**) three white and (**b**) three red wines. Each color represents an aroma descriptor. The arc length/central angle of each pie sector (aroma descriptor) is proportional to the sum of the related odor active value (OAV), and the thickness of each line corresponds to the number of compounds that explain each aroma descriptor (shared compounds). For pie chart representation, the mean between the three replicates was considered.

Finally, for construction of the aroma networks of each wine variety, the OAVs were calculated for each volatile compound and summed together based on shared aroma characteristics, thus they were combined with the bipartite network data (Figure 3). The strategy of summing OAVs based on compounds’ aroma characteristics was already applied to evaluate the aroma volatile compound emissions from beef cattle feces and urine [33] or following land application of swine manure [34]; however, few aroma categories were used, which was a drawback to predict the global aroma emitted by these manures. Therefore, in this work, to construct aroma networks, a total of 71 volatile compounds and 19 aroma descriptors were considered. In the aroma networks, two nodes (wine aroma descriptors) are linked if they share at least one aroma descriptor, and the thickness of the line is proportional to the number of shared compounds [15]. The application of the summed OAVs concept to access the aroma characteristics may not account for possible synergistic or other complex interactive effects [35], however, it may be useful to predict the overall aroma properties of different wine varieties, as shown in Figure 3.

#### 2.2.2. Aroma Fingerprint of the Six Monovarietal Wines

According to the results shown in Figure 3, different aroma networks were found for each wine variety under study. In addition, all the aroma descriptors are connected between them, although the intensity of the aroma descriptors and the number of shared compounds are different. Conceptually, compounds with larger OAVs are assumed the most dominant compound that will contribute to the overall aroma of a complex mixture like wine.

##### Sauvignon Blanc Wine

The aroma network of Sauvignon Blanc wine is characterized by tree fruit, sweet, and flowery notes. These aromas are mainly related to esters, C_6_ alcohols, and thiols that are present at concentrations above their odor threshold (Table S1). Sauvignon Blanc wine exhibited ethyl dihydrocinnamate, hexyl acetate, and phenylethyl acetate in its composition, all described as contributing to tree fruit and sweet aroma descriptors. Sauvignon Blanc wine also exhibited toasted aromas (Figure 3a) and these can be explained by the presence of several thiols, namely 2-methyl-3-furanthiol (OAV 151.6), whose OAV is higher than those of the other white wines (OAVs ranging from ca. 44 and ca. 132 for Arinto and Bical, respectively). Thus, according to these results, thiols, esters, and C_6_ alcohols have an important role in the aroma properties of this wine. This is in line with a previously reported study performed with 79 Sauvignon Blanc wines produced in different countries (New Zealand, United States, South Africa, Chile, Australia, and France) and vintages (2003–2005). Furthermore, as a worldwide cultivated variety, Sauvignon Blanc is reported as a white wine variety that can exhibit different aroma styles, and this is essentially related to the variations in the concentrations of the chemical compounds belonging to the referred chemical families [28].

##### Arinto Wine

According to the aroma network determined for Arinto wine (Figure 3a), higher fractions of tree fruit, flowery, and sweet aroma descriptors were found, sharing 25, 14, and 10 aroma compounds, respectively, with Sauvignon Blanc wine. These are very much related to the higher OAVs determined for esters and C_13_ norisoprenoids, principally for ethyl octanoate (OAV ca. 38), ethyl hexanoate (OAV ca. 22), and β-damascenone (OAV ca. 236). Beyond the fact that β-damascenone was determined in the other white wines (Bical and Sauvignon Blanc), its OAV was higher in Arinto wine than in Sauvignon Blanc (OAV 184) and 3.5 times higher in Arinto than in Bical (OAV 67) wine. This is a compound well known for its contribution to a sweet and flowery aroma. Arinto wine also exhibited low contribution of toasted (some thiols), herbaceous (some alcohols, thiols, and some esters), and oxidized (some acids) aroma perceptions (Figure 3a), which may be explained by the presence of compounds exhibiting lower OAVs (Table S1).

##### Bical Wine

Based on the constructed aroma network, Bical wine is characterized by tree fruit, sweet, and floral notes, as determined for Arinto wine, or even for Sauvignon Blanc (Figure 3a). However, these aromas were less intense when compared to other white wines. This could be explained by the fact that the OAVs, determined essentially for esters and C_13_ norisoprenoids and also for phenylethanol were lower when compared to the other white wines (Table S1). Furthermore, Bical wine also presented higher fractions of oxidized and herbaceous aromas. This can be explained by the higher OAVs determined in this wine for acids [36], alcohols [26,37], and also some thiols [19]. Furthermore, higher tobacco notes related to benzylmercaptan were determined in the aroma network of Bical wine. The OAV (218.34) determined for this compound in Bical wine was ca. 7 and 35 times higher than in Arinto (31.67) and Sauvignon Blanc (7.17) wines, respectively.

For white wines aroma networks, the fractions related to tropical fruit and citric aroma notes were smaller when compared with the previously referred fractions (Figure 3a). Tropical aroma notes are mainly related to the presence of some esters (isoamyl acetate, hexyl acetate), lactones (δ-octalactone, *(E)*-whiskylactone) and thiols (4-mercapto-4-methyl-2-pentanone), while citric ones are related with terpenic compounds. A great part of these aroma compounds exhibited OAVs lower than one (Table S1), which justifies the lower fractions determined on the white wine’s aroma networks. Little contributions were also observed for fermented aromas, which are mainly related to the presence of some acids (isovaleric, octanoic, and decanoic acids) and methionol (Figure 3a). These components present low OAVs, ranging from ca. 0.3 to 9 (Table S1).

##### Baga Wine

Concerning the aroma network of Baga wine, tree fruit and sweet are the predominant aromas (Figure 3b). These are related essentially with ester compounds, principally ethyl octanoate and ethyl hexanoate (both with OAVs higher than 39), and with β-damascenone (OAV 53.31) (Table S2). On the other hand, some lactic, oxidized, and fusel/alcohol aromas were also determined, but with lower fractions when compared with fruity and sweet ones. Lactic and oxidized aromas are mainly explained by the higher OAVs determined for some acids, principally isovaleric, butyric, isobutyric, and octanoic acids, while fusel aromas were mainly explained by the higher OAVs determined for isoamyl alcohol and isobutanol (Table S2). 

##### Castelão Wine

The aroma network determined for Castelão wine is shown in Figure 3b. As stated for Baga wine, Castelão also presented tree fruit and sweet aromas, although their fractions were present in lower amounts. In addition to the fact that these wines share 14 aroma compounds related to these aromas, the lower fruity and sweet aromas of Castelão wine are mainly explained by the lower OAVs (Table S2) determined for ester compounds. Similar to Baga wine, Castelão also exhibited oxidized and lactic aroma perceptions, which are mainly related to acids, whose OAVs determined were very similar to those found in Baga wine. In addition, similar fusel aromas were determined for Baga and Castelão wines related to the higher OAVs determined for isoamyl alcohol and isobutanol. These wines share four fusel aroma compounds. Furthermore, Castelão wine also presents toasted notes, and this is explained by the higher OAV determined for 2-methyl-3-furanthiol (OAV ca. 82).

##### Touriga Nacional Wine

The Touriga Nacional aroma network revealed its high expression of tree fruit aromas (Figure 3b), which can be explained principally by the higher OAVs found for esters and β-damascenone when compared with the other wines under study. Moreover, considering the three contributions from fruity aromas, which include tree, tropical, and berry fruit aromas (Figure 3b), the higher fractions detected in Touriga Nacional wine suggests that this wine exhibits the highest fruity character considering the red wines under study. Touriga Nacional also exhibited sweet and oxidized notes, although these fractions were very similar among the three red wines studied. As stated for Castelão wine, Touriga Nacional presents toasted aromas, mainly explained by the higher OAV determined for 2-methyl-3-furanthiol (OAV ca. 76). Furthermore, flowery aromas, principally related to C_13_ norisoprenoids (β-damascenone and β-ionone) were also characteristic of the aroma network of Touriga Nacional. 

For the red wine aroma networks, the fractions related to spicy aromas were lower when compared with the other fractions. Spicy aromas are related to the presence of some lactones (γ-nonalactone, γ- and δ-decalactone), phenols (guaiacol, eugenol, 4-propylguaiacol, and 4-vinylguaiacol) and also 3-mercaptohexyl acetate. However, a great part of these aroma compounds exhibited OAVs lower than one (Table S2), justifying the lower fractions determined on the red wine aroma networks (Figure 3b). 

#### 2.2.3. New Insights from Aroma Network Analysis

The aroma network approach obtained a holistic view combining the complex wine volatile data with aroma notes of the wines (aroma descriptors) to objectively establish a chemical aroma fingerprint reflecting all the impact aromas of a wine variety. The aroma network approach is a rapid and visual way to determine wine aroma properties that allows to obtain complementary information to the volatile composition data (Tables S1 and S2, heatmap of Figure 1).

According to Figure 3, for the set of the three white wines and three red ones, all the aroma notes are connected between them, however, the intensity of the aroma notes (arc length/central angle of each pie sector) and the number of shared compounds (thickness of each line) are different. The aroma network constructed for Sauvignon Blanc wine (Figure 3a) revealed that this wine is characterized by tree fruit, sweet, flowery, and toasted notes, accounting for aroma intensities of 28%, 24%, 19%, and 13%, respectively, thus representing 84% of the global wine aroma. Arinto wine was also characterized by tree fruit (30%), sweet (26%), and flowery (21%) notes, which represent 77% of Arinto global aroma (Figure 3a). Finally, Bical wine also had these aroma notes (tree fruit (17%), sweet (12%), flowery (9%)), but they only characterized 38% of the global aroma. For Bical wine, tobacco (21%), and herbaceous notes (8%) were also characteristic of this wine. Moreover, a similar number of shared compounds responsible for tree fruit, sweet, and flowery notes were determined for the three white wines, however, these aroma fractions were lower in Bical wine, which is explained by the lower OAVs determined in this wine (Figure 3a).

Concerning the constructed aroma networks for the red Bairrada wines (Figure 3b), Touriga Nacional was essentially characterized by tree fruit (27%) and sweet (18%) aroma notes. Also, toasted (9%), oxidized (9%), and flowery (8%) notes were characteristic of this wine. All these aroma notes explain 71% of the global aroma of this red wine. Castelão wine was characterized by tree fruit (24%), sweet (16%), oxidized (10%), toasted (9%), and fusel (7%) notes, explaining 66% of the whole aroma of this wine (Figure 3b). Baga wine also has tree fruit (25%), sweet (17%), and oxidized (10%) notes, corresponding to 52% of the global wine aroma, however flowery (8%), lactic (8%), and fusel (6%) notes were also a characteristic. Globally, these aroma notes explain 74% of the Baga wine aroma character. Similar shared aroma compounds were determined for all the red wines under study; thus, the aroma specificity of these wines is explained by differences in the OAVs determined for each individual wine component.

### 2.3. Aroma Sensory Analysis

**Figure 4:**
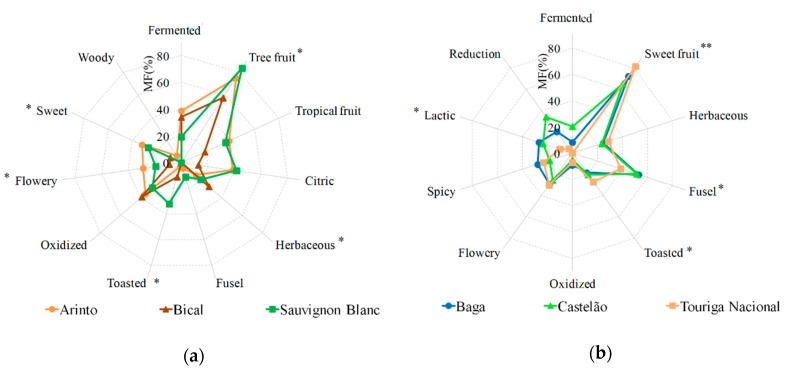
Aroma sensory data expressed as modified frequency (MF) (%) of the six monovarietal wines: (**a**) three white (Arinto, Bical, and Sauvignon Blanc) and (**b**) three red (Baga, Castelão, and Touriga Nacional) wines based on the 11 and 10 sensory terms for white and red wines, respectively, selected by the trained panel (13 judges). To determine discriminant sensory terms ANOVA was applied to the sub-set of white wines and the sub-set of red wines. * *p* < 0.05 and ** *p* < 0.01.

The aroma sensory evaluation of the six monovarietal wines is expressed as modified frequency (MF) (%) and shown in Figure 4. The aroma of the white wines (Figure 4a) is described as fruity (tree and tropical fruits), citric, herbaceous, toasted, oxidized, fermented, flowery, and sweet. Particularly, the worldwide cultivated variety of Sauvignon Blanc wine is characterized by fruity (tree: apple, pear, and tropical: banana, pineapple), sweet, and toasted notes. These sensory descriptions determined by the trained panel follow the constructed aroma network (Figure 3a). Sensory analysis unveiled that the autochthonous Arinto white variety, beyond fruity notes, also exhibited the highest MF (%) values related to flowery and sweet notes (Figure 4a), which follow the aroma network representation (Figure 3a). This may be explained by the higher OAV of β-damascenone determined in Arinto wine compared to the other white wines. Furthermore, some fermented notes were identified by the trained sensory panel, however this note was not a discriminant term among the white wines. The aroma of Bical wine was characterized essentially by tree fruit and herbaceous notes, revealed by both sensory data (Figure 4a) and expressed in the aroma network (Figure 3a).

The aroma of red varieties (Figure 4b) is characterized as sweet fruits, which include tree (apple, pear), tropical (banana, pineapple), and berry fruits (strawberry, raspberry, blackberry), herbaceous, fusel, toasted, flowery, spicy, lactic, reduction, and fermented. Sensory analysis revealed that Touriga Nacional wine exhibited higher MF (%) values related to sweet fruits (including tree, tropical, and berry fruits) and toasted notes. The lower MF (%) values were related to lactic, fusel, and reduction notes, while Castelão and Baga wines had similar MF (%) of these attributes. Furthermore, Baga wine exhibited lactic notes (Figure 4b). The aroma profile described by the panel for the red wines is in line with the aroma notes described by the aroma networks of each red wine (Figure 3b). Moreover, according to ANOVA analysis performed for the sub-set of white and to the sub-set of red wines, the most discriminative terms are tree fruit, sweet, flowery, toasted, and herbaceous notes (*p* < 0.05) for white wines (Figure 4a), and sweet fruits (*p* < 0.01), lactic, toasted, and fusel notes (*p* < 0.05) for the red wines (Figure 4b).

## 3. Materials and Methods 

### 3.1. Wines Under Study

For proof-of-concept of the aroma network approach, six young monovarietal wines (all of them less than one year), three white and three red, with different aroma characteristics from 2010 harvest were studied: *Vitis vinifera* L. *cv*. Sauvignon Blanc (12.5% alcohol, *v/v*), Arinto (12.0% alcohol, *v/v*), Bical (13.0% alcohol, *v/v*), Baga (12.5% alcohol, *v/v*), Castelão (14.0% alcohol, *v/v*), and Touriga Nacional (15.0% alcohol, *v/v*) varieties. The wines were produced in Manuel dos Santos Campolargo, Herdeiros company, from Bairrada Appellation (Portugal). These varieties are recommended for QWPSR (quality wine produced in a specified region) of Bairrada Appellation. Also, Baga is the most cultivated variety in this Appellation and represents 90% of the total red Bairrada vineyard. Vineyards of Arinto and Bical varieties represent 20% (10% for each one) of the total white grapes’ vineyard.

Briefly, the white grape varieties were pressed, decanted, and stored in atmospheric controlled conditions to prevent oxidation effects. For Sauvignon Blanc variety, the fermentation occurred in steel vats (7500 L) with controlled temperature (12 °C) and without the addition of commercial yeasts. For Arinto and Bical varieties, must fermentation occurred without the addition of commercial yeasts, with batonnage in barrels (300 L wood barrels were used), without temperature control. The red grape varieties were destemmed and maceration occurred at cold temperatures (ca. 15 °C) using mechanical pressing. The fermentation occurred without the addition of commercial yeasts and temperature control (not exceeding 30 °C). At the end of fermentation, the wines were transferred to wood barrels (300 L) where malolactic fermentation occurred. All samples were bottled (3 bottles of 0.75 L for each wine variety), sulfated, and stored at 4 °C in the dark until analysis.

### 3.2. Reagents and Standards

Dichloromethane, HPLC quality, was from Fisher Scientific (Loughborough, UK), methanol of LiChrosolv quality was from Merck (Darmstadt, Germany), absolute ethanol (ACS quality) was purchased from Panreac (Barcelona, Spain), and pure water was obtained from a Milli-Q purification system (Millipore, Billerica, MA, USA). LiChrolut EN resins and polypropylene cartridges were obtained from Merck (Darmstadt, Germany). The aroma chemical standards were supplied by Aldrich (Gillingham, UK), Fluka (Buchs, Switzerland), Sigma (St. Louis, MO, USA), Lancaster (Strasbourg, France), PolyScience (Niles, IL, USA), ChemService (West Chester, PA, USA), Interchim (Monlucüon, France), International Express Service (Allauch, France), and Firmenich (Geneva, Switzerland). α,α,α-Tris-(hydroxymethyl)-methylamine (Tris) 99.9% was obtained from Aldrich-España (Madrid, Spain), and cysteine 99% and phydroxymercuribenzoic acid were from Sigma (St. Louis, MO, USA). The chemical standards used were 2-butanol, 4-methyl-2-pentanol, 2-octanol, and 4-hydroxy-4-methyl-2-pentanone, supplied by Merck (Darmstadt, Germany), PolyScience (Miles, USA), and Aldrich (Gillingham, UK), respectively. *n*-Hexane for organic trace analysis (UniSolv) was from Merck (Darmstadt, Germany). Diethyl ether for instrumental analysis and mercaptoglycerol were from Fluka (Buchs, Switzerland). Anhydrous sodium sulfate was for analysis ACS-ISO quality from Panreac (Barcelona, Spain). Ethylenediaminetetraacetic acid disodium salt 2-hydrate (EDTA), L-cystein hydrochloride hydrate 99%, 1,4-dithioerythritol, octafluoronaphthalene 96% (OFN), and 1,8-diazabicyclo [5.4.0]undec-7-ene (DBU) were from Aldrich (Steinheim, Germany). *O*-Methylhydroxylamine hydrochloride purum >98% and 2,3,4,5,6-Pentafluorobenzyl bromide (PFBBr) were from Fluka (Buchs, Switzerland). 4-Mercapto-4-methyl-2-pentanone and 3-mercaptohexylacetate were from Oxford Chemicals (Hartlepool, UK). 2-Furfurylthiol and 3-mercaptohexanol were from Lancaster (Strasbourg, France). 2-Methyl-3-furanthiol and 2-methyl-3-tetrahydrofuranthiol were from Aldrich (Steinheim, Germany). Benzylmercaptan, 2-phenylethanethiol, and 4-methoxy-α-toluenethiol were from Fluka (Buchs, Switzerland). Bond Elut-ENV resins, prepacked in a 50 mg cartridge (1 mL total volume) and semi-automated SPE Vac Elut 20 station were from Varian (Walnut Creek, CA, USA).

### 3.3. Determination of Wine Volatile Components Based on Gas Chromatographic Techniques

**Figure 5:**
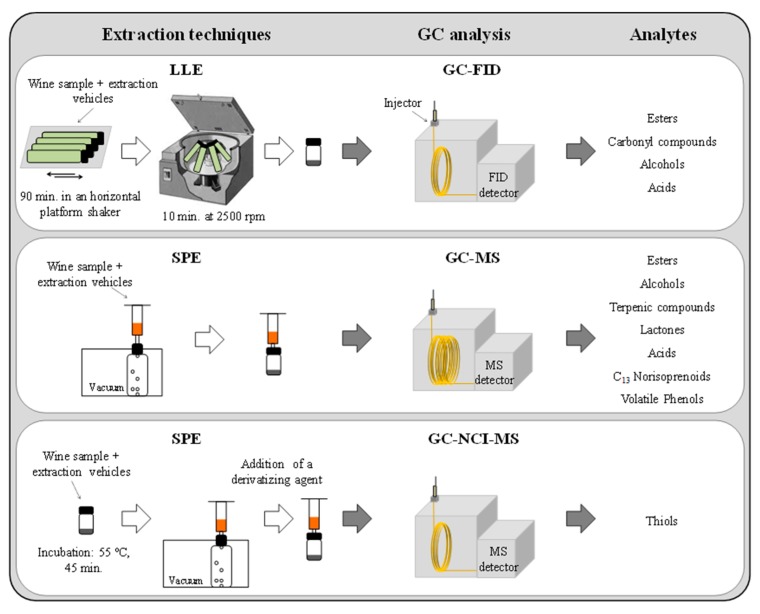
Workflow of the experimental procedures used for the extraction and gas chromatographic analysis for a comprehensive determination of wine volatile composition. LLE: liquid–liquid extraction; GC: gas chromatography; FID: flame ionization detection; SPE: solid-phase extraction; NCI: negative chemical ionization; MS: mass spectrometry.

Figure 5 represents a workflow of the experimental procedures used for the extraction techniques and gas chromatographic analysis for a comprehensive determination of the wines’ volatile composition.

Determination of esters, carbonyl compounds, alcohols, and acids was done by liquid–liquid extraction (LLE)/ gas chromatography–flame ionization detection (GC–FID). Briefly, according to the LLE/GC–FID methodology already proposed [38], 4.1 g of ammonium sulphate ((NH_4_)_2_SO_4_), 2.7 mL of wine, 6.3 mL of water, 20 μL of internal standard solution (2-butanol, 4-methyl-2-pentanol, 4-hydroxy-4-methyl-2-pentanone, ethyl heptanoate, heptanoic acid and 2-octanol at 200 μg/mL in ethanol) and 0.25 mL of dichloromethane were added to 10 mL centrifuge tubes. The tubes were shaken for 90 min, and then centrifuged at 2500 rpm for 10 min, and the dichloromethane phase was recovered (ca. 150 μL). This extract (2 μL) was then analyzed by GC with FID detection. The identification of each volatile compound was confirmed by the coincidence of the retention times of each compound by the corresponding chemical standard. Quantitative data were obtained by interpolation of relative peak areas in the calibration graphs built by the analysis of synthetic wines containing known amounts of the analytes.

- Determination of terpenic compounds, lactones, C_13_ norisoprenoids, volatile phenols, and also some esters, alcohols, and acids was done by solid-phase extraction (SPE)/GC–ion trap–mass spectrometry (MS). According to the previously developed SPE/GC–ion trap–MS methodology [39], 50 mL of wine sample containing 26 μL of surrogate standards solution (isopropyl propanoate, 3-octanone, heptanoic acid, and β-damascone) were passed through a SPE LiChrolut EN cartridge and the retained wine volatile components were eluted with 1.6 mL of 1% methanol dissolved in dichloromethane. Then, an internal standard solution (2-octanol, 4-methyl-2-pentanol, and 4-hydroxy-4-methyl-2-pentanone in dichloromethane) was added to the eluted sample. The extract (3 μL) was then analyzed by GC with ion trap–MS, scanning the range 35–300 *m/z* in a full scan acquisition mode. Identification of each volatile compound was confirmed by the coincidence of the retention times and mass spectra of each compound with the corresponding chemical standard. Quantitative data were obtained by interpolation of relative peak areas in the calibration graphs obtained from the GC–MS of dichloromethane solutions containing known amounts of the analytes.
- Determination of thiols was done using SPE/GC–negative chemical ionization (NCI)–MS. Based on the developed SPE/GC–NCI–MS methodology for the determination of thiols [40], a vial with 25 mL of wine, 0.2 g of ethylenediaminetetraacetic acid disodium salt 2-hydrate (EDTA) (5 g/L), and 0.6 g of L-cysteine clorhydrate (0.1 M Cys) was shaken for 2 min. After this, 15 μL of an ethanolic solution containing the internal standard (1.4 ng/L of 2-phenylethanethiol) was added and shaken to ensure complete dissolution. Then, 0.2 g of *O*-methylhydroxylamine was added to the sample and incubated (55 °C, 45 min). After this, 6 mL of the incubated sample was loaded onto a Bond Elut-ENV SPE cartridge. Then, a second internal standard was also loaded into the cartridge (20 μL of an ethanolic solution containing 150 μg/L of 4-methoxy-α-toluenethiol and 200 μL of water). The thiols retained in the SPE cartridge were directly derivatized by passing 1 mL of an aqueous solution of 1,8-diazabicyclo[5.4.0]undec-7-ene (DBU) (6.7%) and 50 μL of a 2 μg/L solution of 2,3,4,5,6-pentafluorobenzyl bromide (PFBBr) in hexane. Derivatized analytes were eluted with 600 μL of hexane/diethylether (25%), and then an internal standard (1,4-dithioerythritol octafluoronaphthalene (OFN), 22.5 μg/L) was added. This extract (4 μL) was directly injected into the GC–NCI–MS system and the analytes were acquired in single ion monitoring (SIM) mode.

To obtain the concentration data, the corresponding analyte peak relative areas were divided by the slopes determined in the calibration graphs. For each methodology used, each wine sample was extracted in triplicate.

### 3.4. Wine Aroma Sensory Analysis

The sensorial panel was composed of eight females and five males, between the ages of 23 and 68, all of them belonging to the Laboratory for Flavor Analysis and Enology of Zaragoza (Spain), with years of experience in sensorial analysis, statistically proved repeatability, and sample discrimination capacity. Five training sessions (ca. 1 h) were carried out. In the first one, judges generated descriptive terms for Bairrada wines. In sessions 2 and 3, different aroma standards were presented and discussed by the panel, where a total of 15 aroma terms (Table S3) were selected for the following descriptive analysis: 11 for white wines (fermented, tree and tropical fruits, citric, herbaceous, fusel, toasted, oxidized, flowery, sweet, and woody) and 10 for red wines (fermented and sweet fruits which include tree, tropical, and berry fruits), herbaceous, fusel, toasted, oxidized, flowery, spicy, lactic, and reduction). In sessions 4 and 5, panelists scored the intensity of each attribute using a 7-point scale: 0 = no odor, 1 = weak and low intense odor, 2 = clear perceptive and intense odor, 3 = extremely intense odor; half values were allowed. After the training period, each panelist participated individually in one session per day (the number of panelists per session varied from 9 to 12), to evaluate the wine samples. In all cases, wines (20 mL, ca. 20 °C, 2 glasses) were presented in coded, tulip-shaped glasses covered by glass dishes and presented in random order. The data processed was a mixture of intensity and frequency of detection (“modified frequency”(MF)), which was calculated with the following formula [41]:

$$\mathrm{MF}\left(\%\right)=\sqrt{F\left(\%\right)I\left(\%\right)\;}$$

where F(%) is the detection frequency of an aroma attribute expressed as a percentage of the total number of judges (*n* = 13) and I(%) is the average intensity expressed as a percentage of the maximum intensity.

### 3.5. Data Processing

A heatmap representation of the full wine volatile data set (6 wine varieties, a total of volatile components, 3 independent replicates), thus performing a hierarchical cluster analysis (HCA), was constructed using MetaboAnalyst 3.0 (web software, The Metabolomics Innovation Centre (TMIC), Canada) [42]. HCA is an exploratory tool applied to characterize the data set and reveal natural groupings (or clusters) within it, through the representation of a dendrogram (tree diagram) and heatmap. Squared Euclidean distances were used and Ward’s minimum variance was used as the clustering algorithm. 

Based on the wine volatile composition, a bipartite network was built, consisting of two different nodes: one node corresponded to the volatile components determined in the studied wines and the other represented the corresponding aroma descriptors (all aroma descriptors are indicated for each volatile compound). A total of 19 aroma descriptors were found (citric, sweet, woody, flowery, honey, coconut, tropical fruit, tree fruit, berry fruit, fermented, toasted, spicy, fusel/alcohol, vanilla, herbaceous, lactic (cheese, butter), oxidized, reduction (animal, leather), and tobacco) for the wine components determined. These categories were attributed based on an in-house database of wine aroma descriptors, constructed with chemical standards with high purity that were analyzed by GC-olfactometry, at the recognized Laboratory for Flavor Analysis and Enology (University of Zaragoza).

The aroma network of each one of the six monovarietal wines was constructed based on the determined bipartite network and on the calculated OAVs and odor threshold value. The percentage of the OAV of each wine component that contributed to each aroma descriptor was determined and represented in pie charts, establishing a chemical aroma fingerprint for each studied wine. For these representations, the mean between the three replicates was considered. In these pie charts, each color represents an aroma descriptor and the arc length/central angle of each sector (aroma descriptor) is proportional to the quantity (OAV) it represents. The thickness of the line corresponds to the number of shared compounds found in the studied wines.

Aroma sensory data were analyzed by two-way analysis of variance (ANOVA), in which wine varieties and judges were considered as the factors (significance level was determined according to the *p*-value obtained). ANOVA was applied to the sub-set of white wines (Arinto, Bical, and Sauvignon Blanc) and the sub-set of red wines (Baga, Castelão, and Touriga Nacional), and notation * indicate significance at *p* < 0.05 and ** *p* < 0.01. The SPSS software for Windows, version 5.0, from SPSS Inc. (Chicago, IL, USA) was used.

## 4. Concluding Remarks

This work disclosed that the construction of aroma networks combining sensory and chemical data provided by GC analysis is a useful approach to explain wine aroma properties, allowing to define the aroma profile of each wine (aroma ID). Despite the huge importance and effectiveness of the sensory analysis on the evaluation of aroma properties, it is time-consuming and does not provide chemical data about the molecules engaged in the construction of an aroma. In this work, six monovarietal wines, including three white and three red wines, with distinct chemical and sensorial characteristics, were selected to obtain results that were appropriate for the proof-of-concept for this strategy. The construction of the aroma networks includes the combination of sensory and instrumental data and the estimation the wine aroma characteristics by the projection of the volatile composition on the aroma network. Finally, it is important to point out that this approach establishes a chemical aroma fingerprint, which is a simplified and effective way to show each wine aroma profile (aroma ID) established by using both OAV data and the aroma descriptor of each wine component. Furthermore, the obtained information would be the first step in understanding the perception of the impact aroma compounds in a complex aroma mixture, such as wine. This aroma network approach may represent a useful tool in the quality control of wine and/or to estimate its peculiar aroma, notes or off-flavors. Also, this approach can be broadened to several food matrices by constructing and validating specific aroma networks.

## Supplementary Materials

The following are available online, Table S1: Quantitative wine volatile components determination for the three Bairrada white wines studied based on the different GC techniques used, organized by chemical families, odor threshold, content, and odor active value (OAV), Table S2: Quantitative wine volatile components determination for the three Bairrada red wines studied based on the different GC techniques used, organized by chemical families, odor threshold, content, and odor active value (OAV), Table S3: Aroma descriptors used for the sensory descriptive analysis of the Portuguese Bairrada wines studied.
