# Supplementary material for: Revealing the Usefulness of Aroma Networks to Explain Wine Aroma Properties: A Case Study of Portuguese Wines
Source: Molecules. 2020 Jan 9;25(2):272. doi: 10.3390/molecules25020272 (PMC7024348; doi:10.3390/molecules25020272)
Supplement: Supplementary file 1 [file molecules-25-00272-s001.pdf]

Supplementary material

Revealing the usefulness of aroma networks to explain wine aroma properties: the case study of Portuguese wines

Sílvia Petronilho [a], Ricardo Lopez [b], Vicente Ferreira [b], Manuel A. Coimbra [a], and Sílvia M. Rocha[a,*]

[a] QOPNA & LAQV-REQUIMTE, Chemistry Department, University of Aveiro, 3810−193 Aveiro, Portugal

[b] Laboratory for Flavor Analysis and Enology, Instituto Agroalimentario de Aragón (IA2), Department of Analytical Chemistry, Faculty of Sciences, Universidad Zaragoza, Zaragoza, E-50009, Spain

Table S1

Quantitative wine volatile components determination for the 3 Bairrada white wines studied based on the different GC techniques used, organized by chemical families, odor threshold, content, and odor active value (OAV).

		Sauvignon Blanc				Arinto				Bical			
**Compound**	**Odor threshold (μg/L) [a]**	**Content* (μg/L)**			**OAV (n=3) [b]**	**Content (μg/L)**			**OAV (n=3)**	**Content (μg/L)**			**OAV (n=3)**
Esters													
Ethyl acetate (a)	12264	18700.6	19540.8	19120.7	1.56	13422.5	13483.6	13453.1	1.10	12401.2	12458.4	12429.8	1.02
Ethyl propanoate (a)	10	94.1	97.0	95.6	9.56	94.9	92.3	93.6	9.36	103.8	107.7	105.8	10.58
Ethyl butyrate (a)	20	99.5	100.1	99.8	5.00	91.7	96.1	93.9	4.70	68.1	70.1	69.1	3.46
Isoamyl acetate (a)	30	289.8	301.1	295.5	9.85	226.2	231.1	228.7	7.62	18.4	18	18.2	0.61
Ethyl hexanoate (b)	14	293.7	279.9	286.8	20.49	307.6	296.2	301.9	21.57	174	180.9	177.5	12.68
Hexyl acetate (b)	1500	58.6	53.8	56.2	0.04	17.8	17.1	17.5	0.01	n.d.	n.d.	n.d.	_
Ethyl lactate (c)	154636	10892.2	11225.6	11058.9	0.07	8561.4	8428.3	8494.9	0.06	7288.2	6926.6	7107.4	0.05
Ethyl octanoate (d)	5	198.1	201.6	199.9	39.97	193.6	184.2	188.9	37.78	100.1	104	102.1	20.41
Ethyl decanoate (d)	200	58.9	62.3	60.6	0.30	60.3	68.1	64.2	0.32	60.5	69.0	64.7	0.33
Diethyl succinate (e)	200000	359.8	363.6	361.7	0.001	301.3	330.7	316	0.00	245	211.2	228.1	0.00
Phenylethyl acetate (d)	250	244.9	230.7	237.8	0.95	187.4	185.1	186.3	0.75	n.d.	n.d.	n.d.	_
Ethyl isobutyrate (e)	15	17.6	16.9	17.3	1.15	22.5	23.3	22.9	1.53	32.0	34.1	33.4	2.20
Isobutyl acetate (e)	1600	71.5	74.5	73.0	0.05	26.3	25.0	25.9	0.02	46.8	45.1	45.9	0.03
Butyl acetate (e)	1880	0.4	0.4	0.4	0.000	5.2	5.0	5.1	0.00	1.9	1.9	1.9	0.00
Ethyl 2-methylbutyrate (e)	18	4.2	4.1	2.3	0.23	3.0	3.2	3.1	0.18	4.1	4.0	4.1	0.23
Ethyl isovalerate (e)	3	5.1	5.3	5.2	1.74	6.0	6.2	6.1	2.04	4.2	4.1	4.2	1.39
Ethyl furoate (e)	16000	12.5	13	12.8	0.001	10.0	9.9	9.9	0.00	4.1	4.0	4.0	0.00
Ethyl dihydrocinnamate (e)	1.6	0.1	0.1	0.1	0.06	n.d.	n.d.	n.d.	_	0.2	0.2	0.2	0.13
Methyl vanillate (e)	3000	4.7	4.8	4.8	0.002	5.1	4.8	4.9	0.00	6.0	6.3	6.1	0.00
Ethyl vanillate (e)	990	9.8	10.2	10.0	0.01	10.9	10.7	10.7	0.01	8.8	9.2	9.0	0.01

Carbonyl compounds													
Acetaldehyde [f]	500	714.9	712.9	713.9	1.43	824.3	830.1	827.2	1.66	640.3	638.5	639.4	1.28
Diacetyl [f]	100	724.8	711.2	718.0	7.18	733.2	729.7	731.5	7.33	957.7	952.7	955.2	9.56
Acetoin [f]	150000	2200.7	2059.4	2130.1	0.01	2441.6	2773.9	2607.8	0.02	2238.5	2357.3	2297.9	0.02
Phenylacetaldehyde [e]	1	12.6	12.1	12.4	12.35	33.5	32.7	33.1	33.10	25.1	24.0	24.6	24.55
Alcohols													
Isobutanol [f]	40000	11651.2	11986.8	11819.0	0.30	10123.9	11013.7	10568.8	0.27	10292.4	10396.8	10344.6	0.26
1-Butanol [f]	150000	117.1	119.2	118.2	0.00	115.3	121.2	118.3	0.00	144.5	146.4	145.4	0.00
Isoamyl alcohol [f]	30000	16990.6	17318.0	17154.3	0.58	10966.9	10329.1	10648.0	0.36	10338.4	10345.6	10342.0	0.34
1-Hexanol [a]	8000	643.6	678.1	660.9	0.08	422.9	444.4	433.7	0.06	704.0	744.2	724.1	0.09
(Z)-3-Hexenol [a]	400	14.1	16.4	15.3	0.04	13.5	14.4	13.9	0.04	27.7	25.4	26.5	0.07
Benzyl alcohol [e]	200000	107.6	101.6	104.6	0.00	28.1	26.5	27.3	0.00	9.2	10.0	9.6	0.00
Phenylethanol [e]	14000	17561.5	17748.8	17655.2	1.26	20485.0	21527.3	21006.2	1.50	15685.2	15369.3	15527.3	1.11
Terpenic compounds													
Linalool [e]	25	11.6	11.4	11.5	0.46	15.6	15.2	15.4	0.62	10.3	10.0	10.2	0.41
Linalool acetate [e]	unknown	0.2	0.2	0.2	_	0.1	0.1	0.1	_	0.2	0.2	0.2	_
α-Terpineol [e]	250	5.0	4.9	5.0	0.02	8.4	8.1	8.2	0.03	3.6	3.6	3.6	0.01
β-Citronelol [e]	100	2.7	2.9	2.8	0.03	3.2	3.3	3.3	0.03	2.6	2.5	2.6	0.03
Geraniol [e]	20	5.1	5.2	5.2	0.26	6.1	6.3	6.2	0.31	4.4	4.8	4.6	0.23
Lactones													
γ-Butyrolactone [c]	35000	2548.4	2606.0	2577.2	0.07	2373.7	2617.2	2495.5	0.08	2051.7	1996.7	2024.2	0.06
(E)-Whiskylactone [e]	790	1.5	1.4	1.5	0.00	0.7	0.7	0.7	0.00	1.1	1.1	1.1	0.00
δ-Octalactone [e]	400	n.d.	n.d.	n.d.	_	17.8	18.0	17.9	0.05	n.d.	n.d.	n.d.	_

γ-Nonalactone [e]	30	3.3	3.4	3.4	0.11	2.2	2.2	2.2	0.07	3.4	3.2	3.3	0.11
γ-Decalactone [e]	88	301.7	309.4	305.6	3.48	304.2	312.3	308.3	3.51	308.1	316.2	312.2	3.55
δ-Decalactone [e]	386	22.8	21.7	22.3	0.06	47.5	46.6	47.1	0.12	41.9	40.2	41.0	0.11
Acids													
Acetic acid [c]	200000	127332.8	127888.5	127610.7	0.64	100069.0	100445.0	100257.0	0.50	137267.3	145240.8	141254.1	0.71
Isobutyric acid [c]	230	170.0	158.8	164.4	0.72	235.1	239.1	237.1	1.03	347.1	339.3	343.2	1.50
Butyric acid [c]	173	656.1	672.2	664.2	3.84	679.3	725.1	702.2	4.06	643.6	704.4	674.0	3.90
Isovaleric acid [d]	33.4	230.0	257.4	243.7	7.30	278.2	262.9	270.6	8.10	285.0	299.9	292.4	8.76
Hexanoic acid [d]	420	3440.2	3458.3	3449.3	8.21	2406.7	2577.5	2492.1	5.94	2595.7	2789.0	2692.4	6.41
Octanoic acid [d]	500	2103.7	2133.1	2118.4	4.24	2084.9	2072.8	2078.9	4.16	2384.3	2340.9	2362.6	4.73
Decanoic acid [d]	1000	374.0	380.9	377.5	0.38	390.2	340.0	365.1	0.37	291.0	288.1	289.6	0.29
Norisoprenoids													
β-Damascenone [e]	0.05	9.3	9.1	9.2	184.00	11.6	12.0	11.8	236.00	3.3	3.4	3.3	67.00
β-Ionone [e]	0.09	0.3	0.3	0.3	3.33	0.3	0.4	0.4	3.89	0.5	0.5	0.5	5.56
Volatile Phenols													
Guaiacol [e]	9.5	0.1	0.1	0.1	0.01	0.4	0.4	0.4	0.04	0.9	0.9	0.9	0.09
Eugenol [e]	6	0.4	0.4	0.4	0.07	4.0	3.9	3.9	0.66	5.2	4.9	5.0	0.85
o-Cresol [e]	3	0.6	0.6	0.6	0.02	0.7	0.7	0.7	0.02	1.1	1.1	1.1	0.04
m-Cresol [e]	68	0.1	0.1	0.1	0.00	0.4	0.4	0.4	0.01	0.6	0.6	0.6	0.01
4-Ethylguaiacol [e]	33	154.8	149.9	152.4	4.62	201.7	198.5	200.1	6.07	123.5	128.9	126.2	3.83
4-Propylguaiacol [e]	unknown	0.03	0.02	0.03	_	0.7	0.8	0.7	_	0.2	0.2	0.2	_
4-Ethylphenol [e]	440	118.9	117.7	118.3	0.27	616.9	604.1	610.5	1.39	233.8	243.2	238.5	0.54
4-Vinylguaiacol [e]	1100	13.3	14.2	13.8	0.01	3.2	3.2	3.2	0.00	8.1	8.5	8.3	0.01
4-Vinylphenol [e]	180	221.5	213.9	217.7	1.21	135.1	132.0	133.6	0.74	129.1	134.8	131.95	0.74

4-Allyl-2,6-dimethoxiphenol (e)	120	0.4	0.4	0.4	0.00	31.6	32.2	31.9	0.03	34.5	32.0	33.2	0.03
Acetovanillone (e)	1000	27.7	28.8	28.2	0.03	56.2	53.1	54.7	0.06	19.1	18.3	18.7	0.02
Thiols													
Methionol (e)	1000	1703.1	1713.7	1708.4	1.71	1213.5	1250.3	1231.9	1.23	1447.8	1479.1	1463.5	1.47
2-Methyl-3-furanthiol (g)	0.0050	0.703	0.813	0.758	151.60	0.239	0.198	0.219	43.70	0.637	0.687	0.662	132.40
2-Furfurylthiol (g)	0.0004	n.d.	n.d.	n.d.	_	n.d.	n.d.	n.d.	_	0.003	0.003	0.002	7.13
4-Mercapto-4-methyl-2-pentanone (h)	0.0008	0.012	0.019	0.016	19.38	0.019	0.014	0.017	20.63	0.017	0.020	0.018	23.13
3-Mercaptohexyl acetate (h)	0.0042	0.001	0.001	0.001	0.30	n.d.	n.d.	n.d.	0.00	0.002	0.000	0.001	0.23
3-Mercapto-1-hexanol (g)	0.0600	0.047	0.052	0.049	0.83	0.031	0.030	0.031	0.51	0.090	0.093	0.091	1.53
Benzylmercaptan (g)	0.0003	0.002	0.002	0.002	7.17	0.009	0.010	0.010	31.67	0.066	0.065	0.065	218.34

[a] Odor threshold values previously reported in the literature (for mixtures of ethanol/water): Campo et al., 2006 and Gómez-Míguez et al., 2007.

[b] OAV: Odor active value (mean of 3 replicates were presented) - OAV of each replicate was calculated by dividing the determined concentration of each wine component by its odor threshold value.

*concentration of wines volatile components was obtained by dividing the chromatographic area of each volatile component by the area of the corresponding internal standard: (a) 4-methyl-2-pentanol, (b) ethyl heptanoate; (c) 4-hydroxy-4-methyl-2-pentanone; (d). heptanoic acid; (e) 2-octanol; (f) 2-butanol; (g) 4-methoxi-α-toluenothiol; (h) 1,4-dithioerythritol octafluoronaphthalene (OFN). Then, the corresponding analyte relative area was divided by the slope determined in the calibration graphs for each volatile compound (data not shown).

n.d. - not detected.

Table S2

Quantitative wine volatile components determination for the 3 Bairrada red wines studied based on the different GC techniques used, organized by chemical families, odor threshold, content, and odor active value (OAV).

		Baga				Castelão				Touriga Nacional			
**Compound**	**Odor threshold (µg/L)** [a]	**Content* (µg/L)**			**OAV (n=3)** [b]	**Content (µg/L)**			**OAV (n=3)**	**Content (µg/L)**			**OAV (n=3)**
Esters													
Ethyl acetate [(a)]	12264	49262.7	48035.2	48648.9	3.97	56271.9	51631.6	53951.8	4.40	64360.2	61364.1	62862.2	5.13
Ethyl propanoate [(a)]	10	202.9	215.2	209.1	20.91	270.1	300.3	285.2	28.52	242.3	238.9	240.6	24.06
Ethyl butyrate [(a)]	20	168.4	157.2	162.8	8.14	196.7	180.4	188.6	9.43	270.5	295.2	282.9	14.14
Isoamyl acetate [(a)]	30	234.3	214.4	224.4	7.48	514.1	511.7	512.9	17.10	384.6	361.9	373.3	12.44
Ethyl hexanoate [(b)]	14	596.6	546.9	571.8	40.84	591.1	551.3	571.2	40.80	683.9	622.4	403.2	46.66
Ethyl lactate [(c)]	154636	109213.7	110090.0	109651.8	0.71	85691.8	83621.7	84656.8	0.55	77143.7	72395.1	74769.4	0.49
Ethyl octanoate [(d)]	5	267.0	268.8	267.9	53.58	238.3	225.5	231.9	46.39	288.8	293.4	211.1	58.22
Ethyl decanoate [(d)]	200	71.7	74.5	73.1	0.37	70.2	68.0	69.1	0.35	55.1	53.3	54.2	0.28
Diethyl succinate [(e)]	200000	5700.9	5476.4	5588.7	0.03	6268.4	6225.0	6246.7	0.03	3042.0	3181.5	3111.8	0.02
Phenylethyl acetate [(d)]	250	124.6	128.1	126.3	0.51	213.0	199.3	206.2	0.83	147.7	149.4	148.6	0.60
Ethyl isobutyrate [(e)]	15	35.6	35.4	35.6	2.37	44.3	45.4	44.9	2.99	42.0	39.9	41.0	2.73
Isobutyl acetate [(e)]	1600	70.6	70.1	70.4	0.04	88.5	84.0	86.3	0.06	125.7	119.7	122.7	0.08
Butyl acetate [(e)]	1880	7.6	7.2	7.4	0.00	3.5	3.6	3.6	0.00	7.3	7.6	7.4	0.00
Ethyl 2-methylbutyrate [(e)]	18	5.5	5.7	5.6	0.31	6.9	7.0	7.0	0.39	5.1	5.2	5.1	0.29
Ethyl isovalerate [(e)]	3	7.2	7.3	7.3	2.44	9.7	10.2	10.0	3.31	5.0	5.3	5.1	1.72
Ethyl furoate [(e)]	16000	2.1	2.1	2.1	0.00	2.9	2.8	2.8	0.00	1.3	1.3	1.3	0.00
Ethyl dihydrocinnamate [(e)]	1.6	0.4	0.4	0.4	0.24	0.5	0.5	0.5	0.31	0.7	0.7	0.7	0.41
Ethyl cinnamate [(e)]	1.1	0.8	0.8	0.8	0.74	1.3	1.3	1.3	1.18	1.4	1.4	1.4	1.29
Methyl vanillate [(e)]	3000	16.8	17.1	16.9	0.01	32.5	34.8	33.7	0.01	57.8	55.3	56.6	0.02
Ethyl vanillate [(e)]	990	659.8	650.9	655.4	0.67	743.9	763.4	753.6	0.76	1241.1	1174.7	1207.9	1.22

Carbonyl compounds													
Acetaldehyde [(f)]	500	1000.6	1068.9	1034.8	2.07	1015.1	1070.9	1043.0	2.09	556.7	542.0	549.3	1.10
Diacetyl [(f)]	100	846.0	860.6	853.3	8.54	518.2	567.0	542.6	5.43	324.9	297.6	311.2	3.12
Acetoin [(f)]	150000	163.6	173.5	168.6	0.00	97.8	95.6	96.7	0.00	199.3	193.3	196.3	0.00
Phenylacetaldehyde [(e)]	1	14.6	14.0	14.3	14.29	14.3	15.1	14.7	14.71	14.0	15.3	14.7	14.64
Alcohols													
Isobutanol [(f)]	40000	79016.2	79109.2	79062.7	1.98	73644.9	69250.0	71447.4	1.79	63013.2	66004.1	69508.7	1.62
1-Butanol [(f)]	150000	1530.4	1536.6	1533.5	0.01	1187.7	1161.1	1174.4	0.01	1517.1	1384.8	1450.9	0.01
Isoamyl alcohol [(f)]	30000	353652.7	353078.0	353365.4	11.78	312121.0	292658.1	302389.6	10.08	266522.1	255224.0	260873.1	8.70
1-Hexanol [(a)]	8000	1520.5	1483.8	1502.2	0.19	1289.7	1234.8	1262.3	0.16	1432.7	1356.7	1394.7	0.18
(Z)-3-Hexenol [(a)]	400	27.1	29.0	28.1	0.07	45.8	49.5	47.6	0.12	34.6	34.8	34.7	0.09
Benzyl alcohol [(e)]	200000	23.1	21.5	22.3	0.00	73.8	77.6	75.7	0.00	57.5	56.9	57.2	0.00
Phenylethanol [(e)]	14000	81537.0	86042.4	83789.7	5.99	71136.2	76894.0	74015.1	5.29	53554.3	56785.9	55170.1	3.95
Terpenic compounds													
Linalool [(e)]	25	5.4	5.6	5.5	0.22	9.2	9.1	9.1	0.37	30.7	29.2	29.9	1.20
Linalool acetate [(e)]	unknown	0.4	0.5	0.5	_	0.5	0.5	0.4	_	0.4	0.4	0.4	_
α-Terpineol [(e)]	250	2.0	1.9	1.9	0.01	4.8	4.6	4.7	0.02	14.8	15.3	15.1	0.06
β-Citronelol [(e)]	100	8.6	7.9	8.3	0.09	12.0	11.5	11.8	0.12	13.5	12.5	13.0	0.13
Geraniol [(e)]	20	8.3	8.1	8.2	0.41	16.0	16.3	16.1	0.81	41.5	39.4	40.5	2.03
Lactones													
γ-Butyrolactone [(c)]	35000	15236.1	16609.5	15922.8	0.46	14132.7	13245.8	13689.2	0.39	8537.7	8203.2	8370.4	0.24
(E)-Whiskylactone [(e)]	790	0.7	0.7	0.7	0.00	1.0	1.0	1.0	0.00	1.5	1.4	1.4	0.00
γ-Nonalactone [(e)]	30	34.0	35.8	34.9	1.16	39.0	38.1	38.6	1.29	25.1	27.2	26.2	0.88
γ-Decalactone [(e)]	88	662.4	678.0	670.2	7.62	628.0	609.3	618.7	7.03	704.7	701.7	703.2	7.99

δ-Decalactone [e]	386	52.6	56.8	54.7	0.15	45.8	41.6	43.7	0.12	58.8	55.8	57.3	0.15
Acids													
Acetic acid [c]	200000	559820.7	591825.6	575823.1	2.88	675261.9	609621.8	642441.9	3.22	798360.9	734655.1	766508.0	3.83
Isobutyric acid [c]	230	2260.8	2099.5	2180.2	9.48	2118.0	2015.0	2066.5	8.99	2012.8	1975.4	1994.1	8.67
Butyric acid [c]	173	1088.9	1049.5	1069.2	6.18	1290.1	1198.0	1244.0	7.19	1464.1	1386.2	1425.1	8.24
Isovaleric acid [d]	33.4	1369.7	1459.9	1414.8	42.36	1589.7	1447.6	1518.6	45.47	1150.4	1198.5	1174.5	35.16
Hexanoic acid [d]	420	2569.8	2548.7	2559.2	6.10	2408.3	2350.8	2379.5	5.67	2678.7	2594.9	2636.8	6.28
Octanoic acid [d]	500	2312.8	2373.9	2343.3	4.69	1990.7	1893.3	1942.0	3.89	1975.3	1865.1	1920.2	3.84
Decanoic acid [d]	1000	681.7	670.2	676.0	0.68	520.3	505.3	512.8	0.52	408.8	424.9	416.9	0.42
Norisoprenoids													
β-Damascenone [e]	0.05	2.7	2.6	2.7	53.31	2.2	2.1	2.2	43.67	3.0	2.9	2.9	58.25
β-Ionone [e]	0.09	0.6	0.6	0.6	6.83	0.6	0.6	0.6	6.21	0.5	0.5	0.5	5.61
Volatile Phenols													
Guaiacol [e]	9.5	10.5	11.1	10.8	1.14	3.7	4.0	3.9	0.41	6.1	5.8	5.9	0.63
Eugenol [e]	6	21.9	22.1	22.0	3.67	18.1	17.3	17.7	2.96	3.4	3.5	3.4	0.58
o-Cresol [e]	31	4.8	4.5	4.6	0.15	4.6	4.5	4.6	0.15	3.8	3.7	3.7	0.12
m-Cresol [e]	68	1.6	1.7	1.7	0.03	1.4	1.3	1.3	0.02	1.8	1.7	1.8	0.03
4-Ethylguaiacol [e]	33	5.1	5.4	5.3	0.16	1.7	1.6	1.6	0.05	7.2	6.9	7.1	0.22
4-Propylguaiacol [e]	unknown	0.1	0.1	0.1	_	0.1	0.1	0.1	_	n.d.	n.d.	n.d.	_
4-Ethylphenol [e]	440	6.0	5.9	5.9	0.01	5.6	5.3	5.5	0.01	3.7	4.3	4.0	0.01
4-Vinylguaiacol [e]	1100	21.1	20.0	20.6	0.02	8.4	7.9	8.2	0.01	12.7	11.5	12.1	0.01
2.6-Dimethoxyphenol [e]	120	17.3	16.6	17.0	0.03	22.6	21.8	22.2	0.04	20.0	19.7	19.9	0.04
4-Vinylphenol [e]	180	2.4	2.3	2.4	0.01	3.16	3.41	3.3	0.02	1.5	1.2	1.4	0.01
4-Allyl-2.6-dimethoxyphenol [e]	120	13.6	14.1	13.9	0.12	15.1	14.9	15.0	0.13	10.1	10.6	10.4	0.09

Acetovanillone [(e)]	1000	334.4	328.9	331.7	0.33	211.3	215.6	213.5	0.22	278.0	266.8	272.4	0.28
Thiols													
Methionol [(e)]	1000	4123.5	4389.2	4256.4	4.26	3336.8	3324.5	3330.7	3.33	2749.1	2629.5	2689.3	2.69
2-Methyl-3-furanthiol [(g)]	0.0050	0.188	0.196	0.192	38.40	0.388	0.431	0.409	81.90	0.347	0.408	0.378	75.5
2-Furfurylthiol [(g)]	0.0004	0.002	0.002	0.002	4.88	0.002	0.002	0.002	5.00	0.002	0.002	0.002	5.38
4-Mercapto-4-methyl-2-pentanone [(h)]	0.0008	0.022	0.024	0.023	28.75	0.022	0.019	0.021	25.63	0.010	0.012	0.011	13.75
3-Mercaptohexyl acetate [(h)]	0.0042	0.001	0.002	0.002	0.38	0.002	0.002	0.002	0.52	0.001	0.002	0.001	0.30
3-Mercapto-1-hexanol [(g)]	0.0600	0.103	0.106	0.105	1.75	0.102	0.104	0.103	1.72	0.047	0.022	0.035	0.58
Benzylmercaptan [(g)]	0.0003	0.004	0.004	0.004	13.83	0.008	0.010	0.009	29.50	0.003	0.004	0.004	12.33

[a] Odor threshold values previously reported in the literature (for mixtures of ethanol/water): Campo et al.. 2006 and Gómez-Míguez et al.. 2007.

[b] OAV: Odor active value (mean of 3 replicates were presented) - OAV of each replicate was calculated by dividing the determined concentration of each wine component by its odor threshold value.

*concentration of wines volatile components was obtained by dividing the chromatographic area of each volatile component by the area of the corresponding internal standard: (a) 4-methyl-2-pentanol. (b) ethyl heptanoate; (c) 4-hydroxy-4-methyl-2-pentanone; (d). heptanoic acid; (e) 2-octanol; (f) 2-butanol; (g) 4-methoxi-α-toluenothiol; (h) 1.4-dithioerythritol octafluoronaphthalene (OFN). Then. the corresponding analyte relative area was divided by the slope determined in the calibration graphs for each volatile compound (data not shown).

n.d. - not detected.

Table S3
Aroma descriptors used for the sensory descriptive analysis of the Portuguese Bairrada wines studied.

Aroma descriptor	References
Fermented	Yeast/ Beer/ Sulfuric
Tree fruits	Apple/ Pear/ Ripe fruits
Tropical fruits	Banana/ Pineapple/ Mango
Citric	Lemon/ Orange
Sweet fruits	Strawberry/ Raspberry and tree and tropical descriptors
Herbaceous	Herb/ Green
Fusel	Ethanol/ Alcoholic/ Vinous
Toasted	Toasted bread/ Vanilla
Oxidized	Cooked vegetables/ Aldehyde-like/ Meat
Flowery	Roses/ Violets/ Lilacs
Sweet	Caramel/ Candies
Woody	Wood
Spicy	Rosemary/ Basil/ Thyme
Lactic	Yogurt/ Cream
Reduction	Stable / Leather / Horse / Burnt
